# Clinical Characteristics and Mortality of Intensive Care Patients With Intracerebral Hemorrhage and COVID-19: A Retrospective Cohort Analysis

**DOI:** 10.7759/cureus.65853

**Published:** 2024-07-31

**Authors:** Mohamed F Al Gharyani, Hassan A Elashhab, Taha Abubaker, Heba Elzawawi

**Affiliations:** 1 Neurosurgery, University of Benghazi, Benghazi, LBY; 2 Neurology, Benghazi Medical Center, Benghazi, LBY; 3 Medicine, University of Benghazi, Benghazi, LBY; 4 Medicine, Benghazi Medical Center, Benghazi, LBY

**Keywords:** intensive care unit, mortality, clinical characteristics, covid-19, intracerebral hemorrhage

## Abstract

Background: Since the COVID-19 pandemic, many studies have reported severe neurologic effects of the infection on the brain. Intracerebral hemorrhage (ICH) is a particular pathology that can result in these devastating neurologic effects.

Objectives: The primary aim of our study is to investigate the possible difference in the clinical and laboratory characteristics of ICH between patients with positive COVID-19 tests and those with negative tests. The potential effect of this difference on the prognosis of the patients during their stay in the intensive care unit (ICU) is a secondary aim of the study.

Methods: In this retrospective cohort review, our data were collected from the electronic medical database of the Benghazi Medical Center (BMC) for the period from January 2021 to June 2022. We depended mainly on the admission paper information documented by emergency doctors, and mortality was measured depending on the clinical status after discharge from the ICU.

Results: This study included a sample of 72 patients, 34 patients (47.2%) were considered COVID-19 positive, and 38 patients (52.8%) were COVID-19 negative. The difference between groups was significant in ICH score ≥3 (higher in positive patients), INR (lower in positive patients), the incidence of new-onset hypertension (higher in positive patients), location of hematoma (infratentorial in positive patients), and intraventricular hemorrhage (IVH) extension (more in positive patients). Also, COVID-19 was significantly associated with ICH score ≥3 (OR 4.6, 95% CI 1.2 - 18.6, p = 0.03, R^2^ = 0.16), INR (𝛃 = 0.35, 95% CI 0.09 - 0.62, p < 0.003, R^2^ = 0.136), risk of ventilation (OR 14.1, 95% CI 3.5 - 56.9, p < 0.001, R^2^ = 0.26), hydrocephalus (OR 9.41, 95% CI 2.72 - 32.5, p = 0.001, R^2^ = 0.19), infratentorial location (OR 3.7, 95% CI 1.1 - 12.5, p = 0.04, R^2^ = 0.14), IVH extension (OR 3.5, 95% CI 1.2 - 10.4, p = 0.03, R^2^ = 0.09), new-onset hypertension (OR 4.2, 95% CI 1.5 - 11.9, p = 0.007, R^2^ = 0.10), and mortality (OR 4.9, 95% CI 1.6 - 15.3, p = 0.04, R^2^ = 0.15). The difference in survivability between groups was statistically insignificant (X^2^ = 0.41, log-rank, P = 0.53).

Conclusion: The current study demonstrates, with sufficient evidence, that COVID-19 infection causes a significant change in some critical baseline characteristics like INR values, location, and IVH extension that influence the prognosis of ICH in ICU patients.

## Introduction

Intracerebral hemorrhage (ICH) is described as bleeding into the brain parenchyma that may also spread into the ventricles and, in rare cases, the subarachnoid space [1]. Hypertension, smoking, excessive alcohol use, hypocholesterolemia, and drug use are all risk factors for ICH. The risk of ICH also increases with age, male sex, Asian ancestry, chronic renal illness, cerebral amyloid angiopathy (CAA), and cerebral microbleeds (CMBs) [2]. Approximately 10% to 20% of all strokes are caused by ICH, which has higher morbidity and mortality rates than ischemic strokes [2].

In December 2019, the first reported case of coronavirus disease 2019, known as COVID-19 and caused by severe acute respiratory syndrome coronavirus 2 (SARS-CoV-2), was discovered in Wuhan, China [3]. Although COVID-19 infections predominantly affect the respiratory system, they result in viral pneumonia via the binding of the SARS-CoV-2 spike protein to the endothelium's angiotensin-converting enzyme-2 (ACE-2) receptor [4]. However, the medical community has hastened to uncover new systemic signs of the virus beyond SARS. The COVID-19 virus may target the central nervous system (CNS), and early in the pandemic, severe neurologic effects of infections were documented [5]. Intracerebral bleeding is a particular pathology that can result in these devastating neurologic effects. Additionally, it has been demonstrated by many studies that patients who experience cerebral bleeding because of a COVID-19 infection are more likely to require intensive care and mechanical ventilation [6]. 

Many studies have hypothesized or reported the concomitant occurrence of ICH and COVID-19. However, no study has yet investigated the possible effect of COVID-19 on the presenting characteristics of ICU patients, and based on our literature review, the number of published research studies with an observational design that suggests this probable link between ICH and COVID-19 still needs to be increased, as many studies that addressed this possible association were either case series or review articles. Accordingly, we decided to investigate the possible difference in the clinical and laboratory characteristics of ICH between patients with a positive COVID-19 infection and those with a negative test and the potential effect of this difference on the prognosis of the patients during their stay in the intensive care unit (ICU). This article was previously presented as a meeting abstract at the 8th edition of the International Conference on Neurology and Brain Disorders on October 24, 2023.

## Materials and methods

Study design

The current study is designed as a retrospective cohort analysis, and our data were collected from the electronic medical database of the Benghazi Medical Center (BMC) from January 2021 to June 2022. All our data were primarily collected for patients who were admitted to the ICU in the corresponding period for primary or secondary diagnosis (developed after ICU admission) of ICH, and for additional quality control of the study protocol, we reviewed all records with diagnostic entry to the ICU for any medical or surgical condition potentially linked to ICH and assessed its COVID-19 status. This process permitted us to identify all ICH cases in the BMC receiving medical attention in the ICU and helped us reduce the risk of selection bias. The Human Scientific Ethics Committee of Benghazi Medical Center approved the study protocol (No. MNMT-1-44-91-2021) and complied with local regulations and the Declaration of Helsinki. Based on the observational design of the study, the committee approved the current study without needing to obtain informed consent to participate.

Spontaneous ICH is defined as bleeding in the brain tissue due to a rupture of one of the cerebral vessels, where the diagnosis of ICH is confirmed by radiographic imaging by a specialized neurosurgeon and neuroradiologist. COVID-19 infection was confirmed based on a reverse transcriptase polymerase chain reaction (RT-PCR) assay of a respiratory tract sample positive for SARS-CoV-2, along with radiological and clinical confirmation by respiratory specialists.

Study population (inclusion and exclusion criteria)

The main enrollment strategy of the study patients was their diagnosis with ICH either before or during their stay in the ICU (surgical and medical ICU); furthermore, patients with a positive COVID-19 test at admission or up to 28 days prior (as the average time between the onset of initial symptoms and ICH identification was 20.4 days) and who presented with ICH or subsequently developed ICH during their ICU course were all included as ICH patients with a positive COVID-19 (pre-stroke COVID-19 patients), so patients who developed ICH due to any other cause before the confirmation of their COVID-19 infection (post-stroke COVID-19 patients) were excluded from the study to avoid selection biases [7].

Furthermore, patients with a history of trauma, patients less than 18 years old, those with ward admission only, patients diagnosed with cerebrovascular malformation by a specialized neuroradiologist and neurosurgeon, patients on anticoagulant or aspirin usage, and those with a history of ischemic stroke were excluded from our population to avoid confounding biases.

As stated, this study aims to investigate the potential role of COVID-19 in changing the risk factors for spontaneous ICH. Thus, the rationale behind the exclusion of patients with a cerebrovascular malformation is that the cerebrovascular malformation is considered a non-modifiable risk factor, and accordingly, the presence of patients who are complaining of this risk factor would confound our analysis, especially regarding the size of the hematoma and the location. The exclusion of patients with a history of anticoagulant or aspirin usage was to eliminate any kind of cofounding that could interfere with our analysis to study COVID-19-associated coagulopathy (CAC); thus, any changes to the coagulation profile would be directly related to COVID-19 infection.

Data collection

In the current study, the comparison of the mortality and clinical characteristics was mainly between patients with positive and those with negative COVID-19 infection who were complaining of ICH as the main inclusion criteria. For this reason, we depended mainly on the admission paper information documented by the emergency doctors, and the discharge mortality was measured depending on the clinical status after discharge from the general ICU (medical and surgical ICU). The modified Rankin Scale (mRS) was used for measuring disability in discharged patients, so patients with scores of 0, 1, or 2 were considered to have a full restoration of their premorbid state, but other scores of 3, 4, or 5 were considered to have been discharged with a persistent focal deficit. All our cases were double-checked by a professional neuroradiologist and neurosurgeon for any vessel abnormalities through cerebral angiography. The clinical and laboratory characteristic data were obtained at the time of admission or within 24 hours after admission, as those characteristics were used to predict the ICU prognosis in both groups.

Our clinical review form contained information regarding demographics, etiology, comorbidities, ICH and Functional Outcome of Patients With Primary Intracerebral Hemorrhage (FUNC) scores, laboratory findings, radiological imaging, surgical management, history of medications, outcomes, complications, Glasgow Coma Scale (GCS), and blood pressure. 

Statistical analysis

The statistical analysis of our data was calculated using the Stata Software Package Version 17, where the normality of the data was checked using the Skewness-Kurtosis normality test. In our analysis, the categorical data and the nominal data that were converted to categorical data were expressed as counts and percentages. Continuous data are presented as the means and SDs or ranges, or medians and interquartile ranges (IQRs), as appropriate. The variables of the baseline characteristics were compared using the chi-square X^2^ test or Fisher exact test (categorical variables), the Student t-test (for normally distributed continuous data), and the Mann-Whitney U test (for skewed distribution). Univariate and multivariate logistic regression models were adopted to investigate the potential effect of COVID-19 on baseline characteristics and mortality. Survival analysis was calculated using the Kaplan-Meier test with a 95% CI. Survivability was measured from the date of ICU admission to the death of a patient from any cause or to the last follow-up. Additionally, a comparison of the survival rates between the groups was performed using the log-rank test. A two-tailed test was performed, and P < 0.05 was considered statistically significant. 

## Results

In our analysis, a total of 470 patients were admitted to the ICU in the corresponding period; 157 of them had ischemic strokes (33.4%), and 72 patients (15.3%) complained of ICH and were eligible to be included in our analysis. Overall, 34 patients (47.2%) had ICH and a positive COVID-19 infection, and 38 patients (52.8%) were COVID-19 negative. Thirty patients (88.2%) of the eligible COVID-19-positive cases were presented at admission as cases of COVID-19 infection (respiratory symptoms at presentation) and then subsequently developed ICH during their stay in the ICU, whereas four patients (11.8%) were known cases of mild COVID-19 infection and developed ICH within 28 days (neurological symptoms at presentation). In the first category (respiratory symptoms at presentation), the main presenting symptoms were dyspnea in 28 cases (82.4%), fever in five cases (14.7%), and neurological symptoms in one case (2.9%). In the second category (neurological symptoms at presentation), the main presenting symptoms were sudden loss of consciousness in one case (25%) and sudden body weakness in three cases (75%).

Demographic characteristics

**Figure 1:**
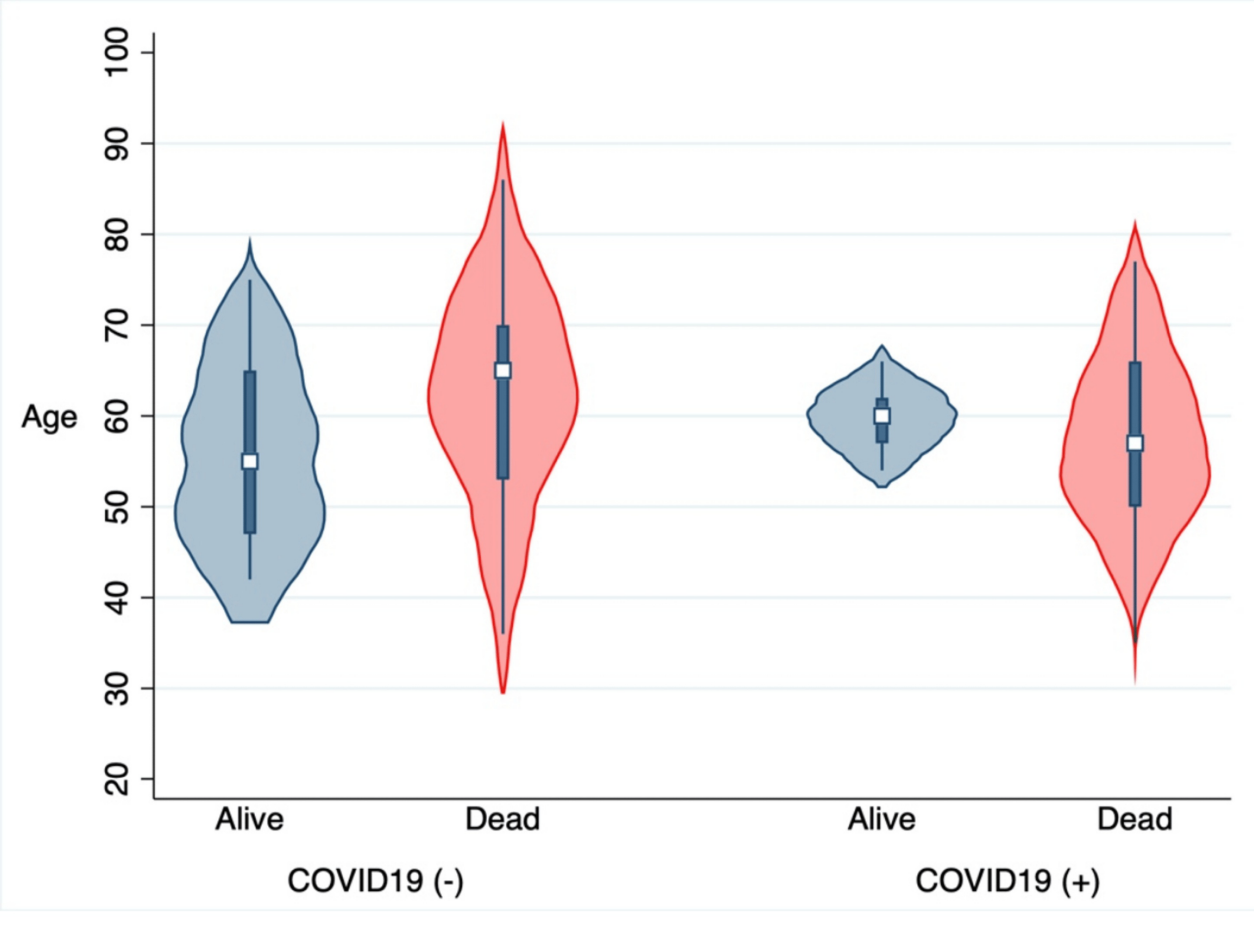
The distribution of age with COVID-19 and mortality Shown is the violin plot that demonstrates a lower median age of mortality for the positive COVID-19 patients with high probability in comparison to those with negative tests. This difference was statistically and clinically insignificant (P > 0.05).

Our study included a series of 46 males (63.9%) and 26 females (36.1%) with an ICH diagnosis (Figure 1). Twenty-seven males (58.7%) had negative COVID-19 infection with a mean age of 57 (SD ± 10), and 19 males (41.3%) had positive COVID-19 infection with a mean age of 58 (SD ± 6). Eleven female patients (42.3%) were COVID-19 negative with a mean age of 61.8 (SD ± 15.5) and 15 female patients (57.7%) were COVID-19 positive with a mean age of 58 (SD ± 10.4). No significant difference was detected between the two groups (P > 0.05); more details are shown in (Table 1), which demonstrates the difference within and between the groups. 

**Table 1:** Baseline characteristics of ICH in patient groups, n = 72 Values represent the number of patients (%) unless indicated otherwise. Values in age, FUNC, lab findings, and SBP represent the number of patients (mean) standard deviation. The boldface type indicates statistical significance. AntiHTN, antihypertensive (ACE inhibitor); Cr, creatinine, GCS, Glasgow Coma Scale; IVH, intraventricular hemorrhage; Ptt, platelets; SBP, systolic blood pressure; FUNC, Functional Outcome of Patients With Primary Intracerebral Hemorrhage; ICH, intracerebral hemorrhage

Variables	COVID-19 (+) (n = 34)		COVID-19 (-) (n = 38)		P-value
	Alive (%) (n = 12)	Dead (%) (n = 22)	Alive (%) (n = 23)	Dead (%) (n = 15)	
Gender					0.18
Male	4 (33.3%)	15 (68.2%)	15 (65.2%)	12 (80%)	
Female	8 (66.7%)	7 (31.8%)	8 (34.8%)	3 (20%)	
Age (yrs)	8 (61.75) ± 5	25 (56.96) ± 9	20 (55) ± 10.8	13 (63.4) ± 12.7	0.9
Ethnicity					0.87
White	10 (83.3%)	20 (90.9%)	19 (82.6%)	15 (100%)	
Black	2 (16.7%)	2 (9.1%)	4 (17.4%)	0 (0%)	
ICH score					0.034
III	0 (0%)	7 (100%)	0 (0%)	4 (100%)	
IV	0 (0%)	1 (100%)	0 (0%)	1 (100%)	
V	0 (0%)	1 (100%)	0 (0%)	0 (0%)	
FUNC	12 (7.92) ± 2.4	21 (7.33) ± 2.3	23 (8.8) ± 2.87	15 (4.8) ± 2.37	0.66
Lab findings					
INR	12 (1.6) ± 0.54	22 (1.7) ± 0.53	23 (1.3) ± 0.2	15 (1.3) ± 0.24	<0.001
Hbg	12 (12.7) ± 2.4	14 (13.6) ± 1.7	19 (13.17) ± 2.7	13 (12.3) ± 3	0.57
Ptt	12 (271) ± 74	22 (322) ± 99	23 (241) ± 59	15 (256) ± 72	0.003
Urea	8 (21) ± 9.3	9 (24.5) ± 12	3 (42.7) ± 45	6 (34) ± 27	0.18
Cr	8 (1.1) ± 0.4	9 (0.97) ± 0.19	3 (1.5) ± 1.2	6 (1.3) ± 0.5	0.06
K	11(4.1) ± 0.4	21 (4.2) ± 0.6	20 (4) ± 0.7	12 (4.4) ± 1	0.8
Size					0.051
>30 mL	5 (41.7%)	6 (27.3%)	17 (74%)	4 (26.7)	
<30 mL	7 (58.3%)	16 (72.7%)	6 (26%)	11 (73.3%)	
Smoking					0.41
Non-smoker	6 (50%)	7 (31.8%)	8 (43.8%)	2 (13.3%)	
Ex-smoker	3 (25%)	10 (45.5%)	7 (30.4%)	5 (33.3%)	
Smoker	2 (16.7%)	4 (18.2%)	4 (17.4%)	8 (53.3%)	
Unknown	1 (8.3%)	1 (4.6%)	4 (17.4%)	0 (0%)	
Ventilation					<0.001
Yes	7 (77.8%)	18 (90%)	4 (17.4%)	10 (66.7%)	
No	2 (22.2%)	2 (10%)	19 (82.6%)	5 (33.3%)	
Hypertension					0.005
Yes	5 (41.7%)	15 (68.2%)	8 (34.5%)	2 (13.3%)	
No	7 (58.3%)	7 (31.8%)	15 (65.2%)	13 (86.7%)	
AntiHTN					0.04
Yes	12 (100%)	21 (95.5%)	0 (0%)	2 (13.3%)	
No	0 (0%)	1 (4.5%)	23 (100%)	13 (86.7%)	
SBP	11 (167) ± 32	19 (180) ± 38	15 (164) ± 19	8 (206) ± 39	0.75
GCS					0.79
15-12	5 (41.7%)	8 (36.4%)	9 (39.13%)	3 (20%)	
11-9	6 (50%)	8 (36.4%)	7 (30.43%)	9 (60%)	
≤8	1 (8.3%)	6 (27.3%)	7 (30.43%)	3 (20%)	
IVH					0.067
Yes	5 (41.7%)	11 (50%)	2 (8.7%)	8 (53.3%)	
No	7 (58.3%)	11 (50%)	21 (91.3%)	7 (46.7%)	
Location					<0.001
Supratentorial	7 (58.3%)	9 (40.9%)	20 (87%)	13 (86.7%)	
Infratentorial	5 (41.7%)	13 (59.1%)	3 (13%)	2 (13.3%)	

Regarding ethnicity, 34 patients (53%) were white with a negative COVID-19 infection, and 30 patients (46.9%) were COVID-19 positive. Concerning black patients, four patients (50%) were negative for COVID-19 infection, and four patients (50%) were positive for COVID-19. No significant difference was detected between the groups (P = 0.87). 

COVID-19 and hypertension

Hypertension was one of the main risk factors for ICH in this study. Thirty patients (41.7%) presented with hypertension, and only four (13.3%) of them had been diagnosed with hypertension. The difference in the distribution of new-onset hypertension was statistically significant between the groups (with/without COVID-19); 20 patients (58.8%) with new-onset hypertension were COVID-19 positive, and 10 patients (26.3%) were COVID-19 negative. Forty-two patients (58.3%) presented without a history of new-onset hypertension; 14 patients (41.2%) were COVID-19 positive, and 28 patients were COVID-19 negative. A statistically significant difference was noted between groups (P = 0.005). In contrast, the difference in mortality was not significant (P > 0.05), and the differences in other comorbidities (Table 2) were statistically significant (P < 0.001), but the difference in mortality was not statistically significant. No significant difference was detected between our groups regarding the presenting systolic blood pressure (p > 0.05), the positive COVID-19 patients presented with SBP = 175.5 (SD ± 35.8), and those with negative tests presented with SBP = 178 (SD ± 33.3). Additionally, no significant difference in mortality was noted between the groups (p = 0.13). 

**Table 2:** Comorbidities distribution of ICH in patient groups, n = 72 CHD: chronic heart disease; CKD: chronic kidney disease; CLD: chronic liver disease; COPD: chronic obstructive pulmonary disease; DM: diabetes mellites; ICH, intracerebral hemorrhage

Co-morbidities	COVID-19 (+) (n = 34)		P-value	COVID-19 (-) (n = 38)		P-value
	Alive (%) (n = 12)	Dead (%) (n = 22)	p = 0.19	Alive (%) (n = 23)	Dead (%) (n = 15)	p = 0.54
Hypertension	2 (16.7%)	1 (4.55%)		0 (0.0%)	0 (0.0%)	
COPD	1 (8.33%)	3 (13.6%)		0 (0.0%)	0 (0.0%)	
CHD	1 (8.33%)	8 (36.4%)		0 (0.0%)	0 (0.0%)	
CKD	1 (8.33%)	1 (4.55%)		2 (8.7%)	0 (0.0%)	
CLD	2 (16.7%)	0 (0.0%)		0 (0.0%)	0 (0.0%)	
DM	1 (8.33%)	0 (0.0%)		1 (4.35%)	1 (6.67%)	
Combined co-morbidities	0 (0.0%)	1 (4.55%)		1 (4.35%)	0 (0.0%)	
NO	4 (33.3%)	8 (36.4%)		19 (82.6%)	14 (93.3%)	

ICH and FUNC score characteristics

In our study, we used ICH and FUNC scores as predictors for ICU mortality for both groups, where the ICH score was "0" in 10 patients (90.91%) with negative COVID-19 and in one patient (9.09%) with positive COVID-19. The score was "1" in 15 patients (62.5%) with negative COVID-19 and in nine patients (37.5%) with positive COVID-19. The score was "2" in eight patients (36.4%) with negative COVID-19 and in 14 patients (63.6%) with positive COVID-19. The score was "3" in four patients (36.4%) with negative COVID-19 and in seven patients (63.6%) with positive COVID-19. One patient with negative COVID-19 (50%) and one patient with positive COVID-19 had a score of "4." The difference was statistically significant between groups (P = 0.03, X^2^ = 12). 

The mean FUNC score was 7.2 (SD ± 3.3) in 38 patients (53.5%) with negative COVID-19 and 7.5 (SD ± 2.3) in 33 patients (46.5%) with positive COVID-19; no significant difference was detected between the groups (P = 0.66, t = -0.45). 

Laboratory findings

The laboratory results of our groups did not show any significant difference, especially for hemoglobin levels, white blood cells, and blood sugar (P > 0.05). Furthermore, the basic metabolic profile, which includes serum electrolytes, urea, and creatinine, was statistically insignificant regarding any potential difference between the groups (P > 0.05). More details regarding these findings are depicted in Table 1. 

The most remarkable result to emerge from the data is the statistically significant difference in the coagulation profile between our groups, where the mean INR in 34 positive COVID-19 patients (47.2%) was 1.6 (SD ± 0.09) and 1.3 (SD ± 0.03) in 38 COVID-19 negative patients (52.78%) and the P-value for the test was statistically significant (P < 0.001, t = - 3.15). The platelet count was 247 (SD ± 64) in 38 negative patients and 304 (SD ± 93) in 34 patients with positive COVID-19 results (P = 0.003, t = - 3.06).

Radiological characteristics

The radiological characteristics of the ICH included the size of the hematoma (>30 cm^3^, ≤30 cm^3^), location (supratentorial or infratentorial), and intraventricular hemorrhage (IVH) extension. The analysis did not reveal any significant difference between the two groups regarding size (P > 0.05), but the difference in location and IVH extension between the two groups were noteworthy; the location was supratentorial in 15 patients (44.12%) with positive COVID-19 and 33 patients (86.8%) with negative COVID-19 infection and infratentorial in 13 patients (38.2%) with positive COVID-19 and five patients (13.2%) with negative COVID-19 infection (P < 0.001, X^2^ = 16.1). 

IVH was noted in 16 patients (61.5%) with a positive COVID-19 infection in comparison to 10 patients (38.5%) with a negative COVID-19 infection (P = 0.067, X^2^ = 3.35). 

ICH complications

In our analysis, we included the difference in complications between the two groups. These complications included epilepsy, which was diagnosed in one patient with a negative COVID-19 infection, pneumonia or ARDS in one patient (14.3%) with a positive COVID-19 infection, and in six patients (85.7%) with a negative COVID-19 infection. Moreover, pulmonary embolism was noted in one patient with a positive COVID-19 infection, and the neurological deficit that was evaluated based on mRS was observed in five patients (38.5%) with a positive COVID-19 infection and in eight patients (61.5%) with a negative COVID-19 infection. Brain herniation was clinically and radiologically diagnosed by an expert neurosurgeon in six patients (85.7%) with positive COVID-19 infection and in one patient (14.3%) with negative COVID-19 infection; the difference was statistically significant between the groups (P = 0.013, X^2^ = 16.1). 

The single most marked observation was hydrocephalus, which was diagnosed radiologically by a specialized neurosurgeon in 18 patients (75%) with positive COVID-19 infection and in six patients (25%) with negative COVID-19 infection (P < 0.001, X^2^ = 12.6). 

ICH outcome

**Figure 2:**
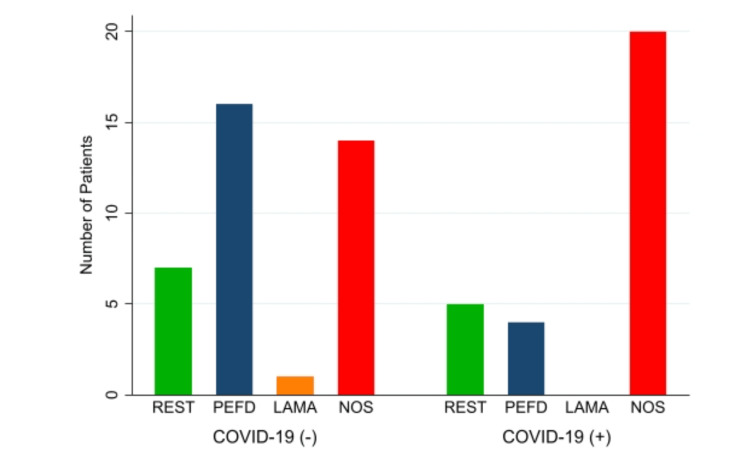
The distribution of clinical outcomes with COVID-19 and mortality Shown is a bar graph that demonstrates the difference in outcome between the two groups; higher mortality was observed in positive cases. LAMA, leave against medical attention “including the transfer to another hospital”; NOS, non-survivor; PEFD, persistent focal deficit; REST, restore to premorbid state

The most intriguing difference in our model was the difference in outcome between our groups (Figure 2), where 12 patients (17.14%) were discharged from ICU with full restoration of premorbid state (restore to premorbid state (REST)); these patients included six patients (50%) with negative COVID-19 infection and six patients (50%) with positive COVID-19 infection. 

Interestingly, 37 patients (52.9%) were non-survivor (NOS) in our study (Table 3), 22 of them (59.46%) were COVID-19 positive, and 15 of them (40.54%) were COVID-19 negative. The persistent focal deficit was detected in 20 cases (28.57%), where four cases (20%) were COVID-19 positive and 16 cases (80%) were COVID-19 negative. The difference was statistically significant between groups (P < 0.05, X^2^ = 7.97). 

**Table 3:** ICH management and clinical outcome data The boldface type indicates statistical significance. DC, decompressive craniectomy; EVD, extra-ventricular drainage; LAMA, leave against medical attention “including the transferring to other hospital”; ME, microscopic evacuation; NOS, non-survivor; PEFD, persistent focal deficit; REST, restore to premorbid state; ICH, intracerebral hemorrhage

Variables	COVID-19 (+) (N = 34)		COVID-19 (-) (N = 38)	P-value
Management				
EVD	10 (29.4%)		2 (5.3%)	0.004
ME	3 (8.82%)		2 (5.3%)	
Craniotomy	7 (20.6%)		3 (7.9%)	
DC	1 (2.9%)		1 (2.6%)	
No intervention	11 (26.8%)		30 (78.95%)	
Clinical outcome				
REST	6 (18.75%)		6 (15.8%)	0.03
PEFD	4 (12.5%)		16 (42.1%)	
NOS	22 (68.75%)		15 (39.5%)	
LAMA	0 (0%)		1 (2.6%)	

Univariate analysis of COVID-19 and clinical characteristics

The unadjusted OR estimate, 95% CI, and P-values for the univariate logistic regression models of COVID-19 (yes/no) versus each baseline characteristic predictor of interest are presented in Table 4. In our study population, COVID-19 was interestingly associated with changes in INR values, ventilation risk, hydrocephalus, infratentorial location, ICH score, and mortality. 

**Table 4:** Univariate and multivariate regression analysis of potential effect of COVID-19 on ICH characteristics The variables INR and SBP were considered continuous variables, and the effect size was expressed as regression coefficients (𝛃). The boldface type indicates statistical significance. ICH, intracerebral hemorrhage

Variables	Crude analysis		Adjusted analysis	
	OR/𝛃 (95% CI)	P-value	OR/𝛃 (95% CI)	P-value
ICH score ≥3	2.75 (0.83 - 9.1)	0.097	4.6 (1.15 - 18.6)	0.03
Hypertension	4.0 (1.48 - 10.8)	0.006	4.2 (1.47 - 11.9)	0.007
INR	0.34 (0.16 - 0.53)	<0.001	0.37 (0.18 - 0.56)	<0.001
IVHe	2.5 (0.93 - 6.68)	0.07	3.5 (1.17 - 10.43)	0.025
SBP	-3.1 (-22.4 - 16.2)	0.75	2.3 (-18.6 - 23.21)	0.83
Size	2.58 (0.99 - 6.76)	0.053	2.7 (0.99 - 7.19)	0.053
Ventilation	10.7 (3.1 - 37.2)	<0.001	14.1 (3.5 - 56.9)	<0.001
Hydrocephalus	6.85 (2.2 - 21)	0.001	9.41 (2.72 - 32.53)	<0.001
Infratentorial	8.4 (2.6 - 26.6)	<0.001	3.7 (1.1 - 12.5)	0.04
Mortality	2.8 (1.1 - 7.3)	0.03	4.91 (1.58 - 15.27)	0.006

Multivariate analysis of COVID-19 and clinical characteristics 

The multivariate predictive model between COVID-19 (yes/no) and the clinical characteristics listed in Table 4 was formulated as multiple binary logistic regression that was adjusted for the clinically critical confounding factors, including age, gender (male), ethnicity, and history of smoking as those factors were non-modifiable by COVID-19 infection. The respiratory component was excluded from the adjustment as most of our cases were complaining of severe respiratory complications, in addition to the absence of a significant correlation between the severity of COVID-19 infection and the severity of intracranial hemorrhage but an additional careful analysis was performed on ventilated cases in both groups to isolate the additional risk of COVID-19 on mortality. The adjusted model demonstrated a potential statistically significant association between the COVID-19 infection and the changes that occurred in some clinically important determinants of the ICH prognosis; these determinants included ICH score ≥3 (OR 4.6, 95% CI 1.2 - 18.6, p = 0.03, R^2^ = 0.16), INR ( r = 0.35, 95% CI 0.09 - 0.62, p = 0.009, R^2^ = 0.136), risk of ventilation (OR 14.1, 95% CI 3.5 - 56.9, p < 0.001, R^2^ = 0.26), hydrocephalus (OR 9.41, 95% CI 2.72 - 32.5, p = 0.001, R^2^ = 0.19), infratentorial location (OR 3.7, 95% CI 1.1 - 12.5, p = 0.04, R^2^ = 0.14), IVH extension (OR 3.5, 95% CI 1.2 - 10.4, p = 0.03, R^2^ = 0.09), new-onset hypertension (OR 4.2, 95% CI 1.5 - 11.9, p = 0.007, R^2^ = 0.10), and mortality (OR = 4.9, 95% CI 1.6 - 15.3, p = 0.04, R^2^ = 0.14).

Survival analysis 

**Figure 3:**
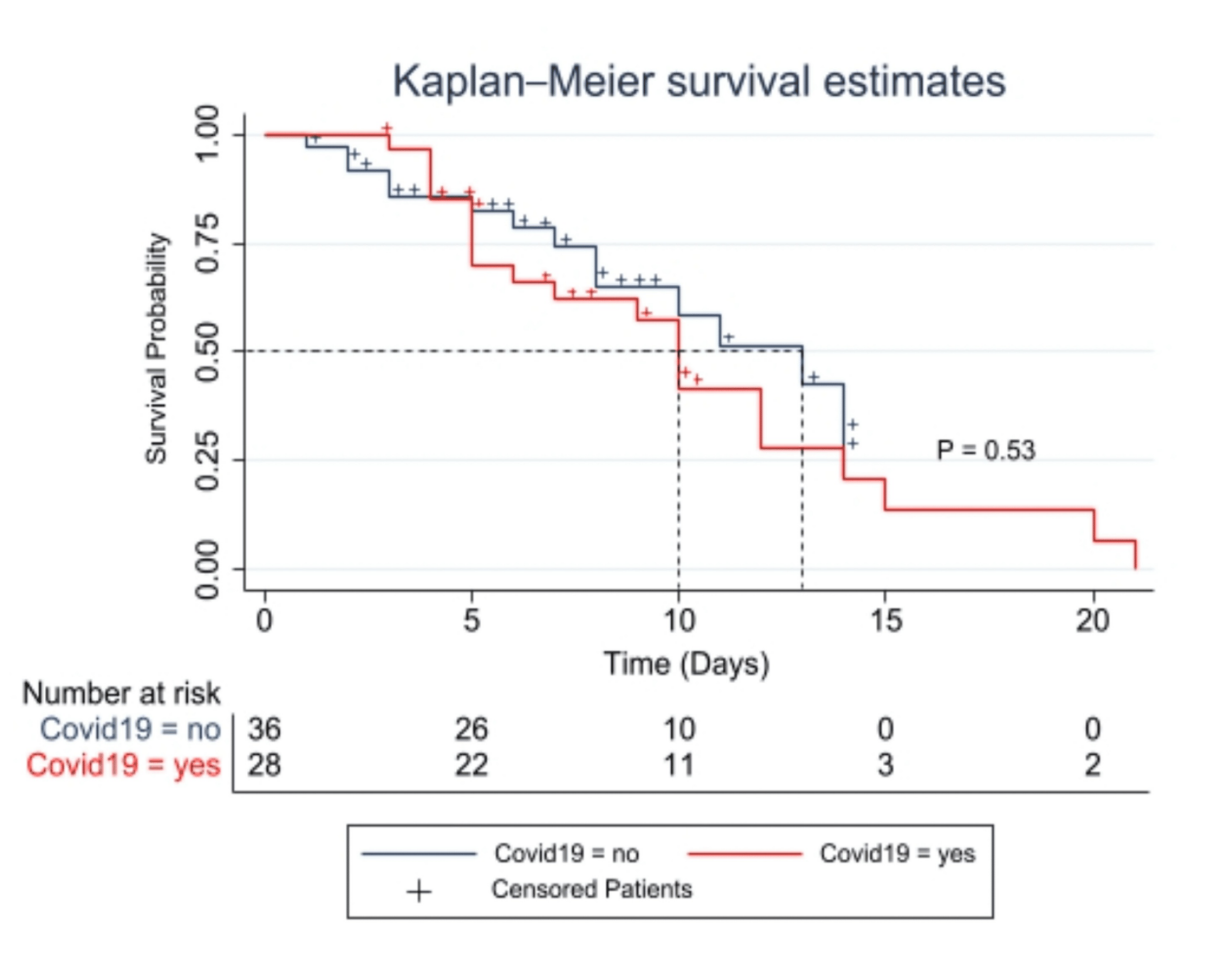
Kaplan-Meier survival analysis comparison curves in patients with ICH with/without COVID-19 infection Shown is the Kaplan-Meier survival estimate of the time from ICU admission to death or ICU discharge. The noted difference in the median survivability between the two groups was clinically and statistically not significant (X^2^ = 0.41, log-rank, P = 0.53).

We analyzed the overall survival (OS) in patients with or without COVID-19 infection (Figure 3). The maximum length of ICU stay for COVID-19-positive patients was 21 days, with a median of eight days, whereas negative COVID-19 patients had 14 days as the maximum length of stay with a median of seven days. The difference in OS between the two patient groups was statistically insignificant (X^2 ^= 0.41, log-rank, P = 0.52). We also examined the potential additional risk of COVID-19 to the OS of patients with ICH. The analysis included some clinically important factors, including age, gender, ethnicity, and history of smoking. Cox regression multivariate analysis for COVID-19 did not show any significant effect on overall survivability. 

## Discussion

**Figure 4:**
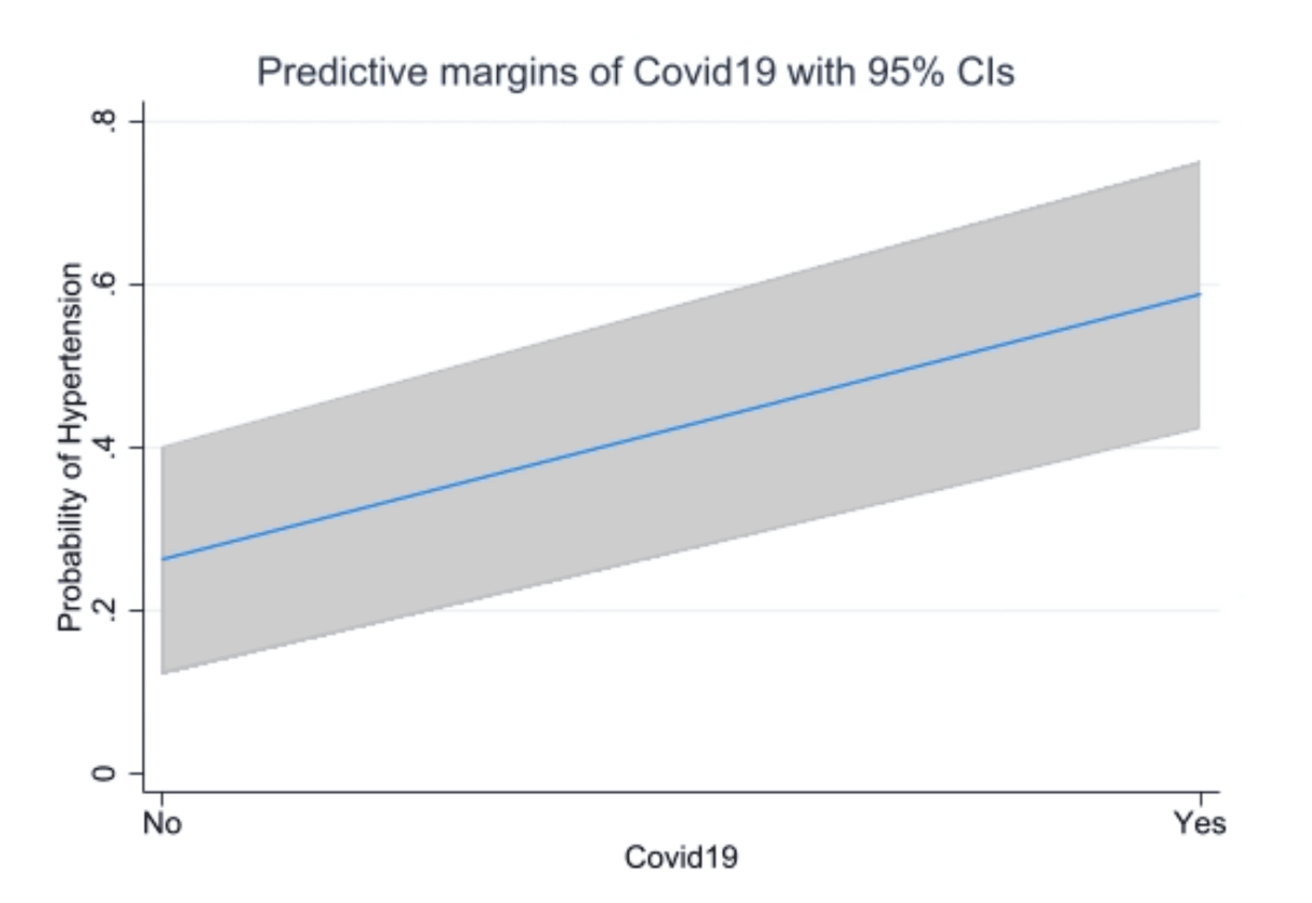
Predicted probability of new-onset hypertension after COVID-19 infection with 95% CI Shown are the predicted margins of the probability of new-onset hypertension between the two groups; the margins graph shows an increased probability of getting new-onset hypertension after COVID-19 infection.

Based on our literature review, the number of published studies on the possible association between COVID-19 and ICH in observational design is insufficient, where most of the published literature was either a case series or review articles or, in the most relevant form, studying intracranial lesions in general [8-10]. The results from the current study demonstrated that ICH is not a common complication of COVID-19 infection in ICU patients compared to ischemic stroke, consistent with other studies [8]. Although we spotted a lower median age with a large probability of dead patients with COVID-19 infection in our plot (Figure 1), this difference was clinically and statistically not significant (P = 0.9), in contrast to earlier findings where they revealed lower ages for the positive patients [9]. Furthermore, no significant difference was detected between males and females in age distribution (P = 0.89) or mortality (P = 0.18). Most of our patients were white (88.9%), but inexplicably only two cases of mortality were detected among black patients with the same ICH characteristics. For the ICH score, which represents the score at ICU admission, the difference was significantly different between the two groups (P = 0.03) as more of the COVID-19 patients (26%) presented with higher scores (≥3) with 30-day mortality exceeding 72% in comparison to 13.2% with the negative test. This finding fits previous findings in the literature and can be explained by the potential indirect effect of COVID-19 on ICH score predictors such as infratentorial location and IVH extension [9]. Conversely, the FUNC score did not reveal any statistically significant difference between the groups (P = 0.66), because only one predictor, "infratentorial location," was different between the groups in contrast to two factors in the ICH score, so we recommend using the ICH score for the prognosis of mortality in ICU patients with positive COVID-19. The laboratory findings of the ICU patients did not reveal any significant difference, with an interesting finding regarding the values of INR in both groups. These values revealed a statistically significant difference between the groups (P = 0.003), without any significant difference between the subgroups (NOS and survivors), and despite this significant finding, the difference was not clinically significant, as the mean difference did not exceed the range of 0.3. Alongside the significant finding regarding the reduced level of platelets between the groups (P = 0.008), it represents an inconsistent finding that can be explained by CAC, which has been stated as an early coagulation dysfunction, characterized by a hypercoagulable and thrombocytopenic state [7,11-13]. This coagulation dysfunction, along with the hypertension status that was observed in 58.8% of COVID-19 patients who were taking ACE inhibitors as treatment for their hypertension, could support the other findings regarding the possible association between COVID-19 infection and ICH development [14]. This study demonstrated a possible relationship between COVID-19 and the new onset of hypertension, with a predicted risk of 58.7% of developing new-onset hypertension after COVID-19 infection (Figure 4).

It is also interesting to note that only n=4 of them were diagnosed with hypertension and taking ACE inhibitors as treatment; no significant difference was detected in mortality in this group, but for those without ACE inhibitors, a statistically significant difference was noted in the presentation of SBP between dead and alive patients (p < 0.05). This result was recorded in good agreement with previous findings in the literature regarding this association [15-21]. Furthermore, the difference in the distribution of comorbidities was statistically significant between groups (p < 0.001) where most of the COVID-19 patients complained of these comorbidities (Table 2), some of which affected the susceptibility of the patients to the infection by either affecting immunity and thus increasing the risk of infection, such as in patients with chronic kidney disease (CKD) (6%) or chronic liver disease (CLD) (6%) or inducing a hypertension-like state in CKD (6%). The effect of heart disease was caused by the use of an anticoagulant that was observed in three patients with positive infections [10,20,22]. The radiological findings that were observed in our cohort did not show any significant difference between our groups regarding the size of the hematoma (P = 0.051), and this is due to the direct relation of the size of the hematoma to the value of SBP at presentation and anticoagulant usage. As our patients were not on anticoagulant usage and the difference in SBP was not significant, we could not detect any significant difference regarding size. Additionally, the association between COVID-19 and the size of the hematoma was statistically insignificant; rather, the difference in location and IVH extension were noteworthy, although the difference was not statistically significant for IVH extension, which was observed in a study of a case series of 19 patients [9]. The crude and adjusted logistic regression models revealed a significant association between IVH extension and COVID-19 infection. Moreover, our comment regarding the surprising finding of infratentorial favoring ICH in COVID-19 patients may be explained by the direct invasion of the virus of the adjacent infratentorial areas through the peripheral nerves like the olfactory nerve, which then migrate through intracellular mechanisms to the adjacent infratentorial areas, as was observed and proposed in case reports and literature review papers [7,23,24]. The difference in complications between groups was significant (p = 0.013). However, the most interesting complication was hydrocephalus (p < 0.001). This complication demonstrated a statistically significant association with COVID-19 infection. This association can be linked to the potential effect of COVID-19 on intraventricular extension, which eventually led to the communicating hydrocephalus that was observed in our cases (66.7%) or herniation caused by infratentorial hemorrhage (33.3%). This observation is supported by the difference that was detected in the management procedures that showed a prevalent use of extra-ventricular drainage (EVD) as a main surgical intervention in the infected cases (p = 0.008). The risk of ventilation in our cohort was statistically significant between our groups (p < 0.001), and as expected, this risk was significantly associated with COVID-19 infection. Thus, this important factor was one of the determinants of mortality in ICU patients that was caused either by respiratory complications or reduced GCS that necessitated immediate intubation. To investigate the isolated increase in the risk of mortality in ventilated patients with/without COVID-19, careful further analysis was applied, taking into account the effect of the interaction between the two factors. The risk was increased from 67.7% in a ventilated patient without COVID-19 to 79.6% in a ventilated patient with COVID-19; thus, this comparison elaborates the additional risk (12%) of mortality that is added by COVID-19 infection in ICU-intubated patients [25,26]. Accordingly, these differences collectively changed the intensive care outcome as the difference was statistically significant, and mortality was higher in the positive patients than in the negative patients who complained of a persistent deficit (Figure 2), as most were presented with survivability characteristics. The increased ICU mortality in COVID-19-positive patients can be attributed to the unfavorable changes that occurred due to the indirect effect of COVID-19 infection, which altered some of these important determinants of mortality [9,14]. 

Contrary to our expectation, the overall survivability was not significantly different between our groups (Figure 3), which indicated that both groups would have a closer duration in the ICU as survivability depended mainly on the type of intervention rather than presenting characteristics. According to this interpretation, the primary function of the different types of intervention was preventing severe complications in ICH, which controls survivability more than the outcome. These important findings of our review must be treated with extreme caution given that our findings are based on a limited number of observations, and thus the results from such analyses should be confirmed by further prospective studies where the control of confounding factors would be more feasible.

Limitations and strengths

The key strength of this study is represented through its method, where the data searching depended on the double-check procedure by two independent study participants. This technique permitted us to identify any case (primary or secondary) potentially linked to ICH receiving management in the ICU. Additionally, the exclusion criteria included patients with a history of ischemic stroke, cerebrovascular malformation, or anticoagulant usage, as most of the other studies did not exclude, so any change in the coagulation profile would be significantly linked to COVID-19.

Conversely, it is plausible to mention the main limitations of this study. The first limitation was the sample size, and because the concomitant ICH and positive COVID-19 cases were infrequent, this limited our capability to formulate a regression model with sufficient controlling factors. Only four factors were used in our model to adjust for the potential confounders, so we could keep the event per variable (EPV) ratio above 10 to avoid any potential risk of overfitting in our model. Additionally, the second notable limitation was the retrospective design of the study, which limited our work in collecting sufficient information, especially regarding laboratory findings, ventilation parameters, and doses of medical treatment. Most of these data were missing completely at random for variables that were investigated in our study, and regression amputation was utilized to restrict the effect of these missing data on our model.

## Conclusions

Depending on the presenting baseline characteristics, the current study demonstrates, with sufficient evidence, that COVID-19 infection causes a significant change in some critical baseline characteristics, including coagulation markers, location, IVH extension, and risk of new-onset hypertension, influencing the prognosis of ICH in ICU patients. Additionally, it indicates the significant association between these critical baseline characteristics and the difference in mortality, as received management in the ICU only changes the duration of stay rather than mortality.
